# AIKON: a modular computer vision platform for historical corpora

**DOI:** 10.1007/s10032-026-00581-x

**Published:** 2026-06-30

**Authors:** Ségolène Albouy, Somkeo Norindr, Paul Kervegan, Fouad Aouinti, Rémy Delanaux, Clara Grometto, Robin Champenois, Stavros Lazaris, Alexandre Guilbaud, Matthieu Husson, Mathieu Aubry

**Affiliations:** 1 LIGM, ENPC, IP Paris, Univ Gustave Eiffel, CNRS, Marne-la-Vallée, France; 2 LTE, Observatoire de Paris-PSL, CNRS, Paris, France; 3 UMR 8167, Collège de France, Sorbonne Université, CNRS, Paris, France; 4 IMJ-PRG, UMR 7586, Sorbonne Université, CNRS, Université Paris-Cité, Paris, France

**Keywords:** Digital humanities, Historical document analysis, Web platform, Computer vision

## Abstract

Historical document analysis has progressed to a point where the main bottleneck for many historical applications is not algorithms, but relevant interfaces, that can support historians’ workflow. While specialized tools exist for text processing and image search, we argue the community lacks a versatile collaborative platform enabling historians to analyze their own corpora from a particular perspective. As a step in this direction, we present aikon , a modular web-platform designed to empower historians with computer vision tools. aikon implements a complete workflow for historical document analysis, from corpus constitution to ai outputs validation and interpretation. It provides a comprehensive research environment combining source management tools with automated processing capabilities as well as multi-user validation and visualization interfaces. We showcase the potential of aikon by presenting modules enabling the investigation of graphical content transmission across large and diverse corpora, a problem for which few modern tools exist, and which is of key interest, for example in History of Science.

## Introduction

**Fig. 1:**
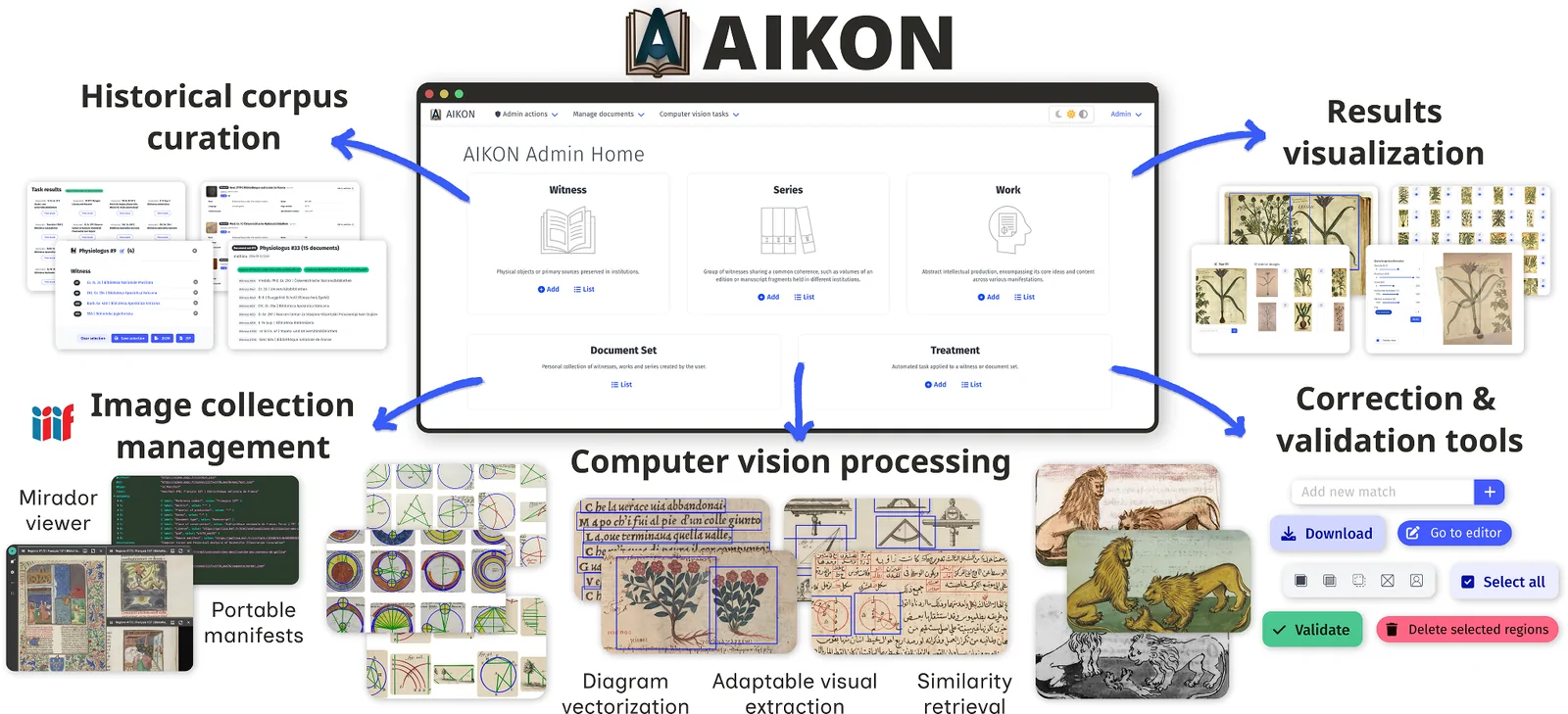
aikon enables to build historical image corpora, apply computer vision algorithms, and curate and visualize the results

We present AIKON, a modular platform designed to foster historians’ collaborations on custom document corpora of digitized historical documents containing images, and support their workflow by leveraging computer vision processes. We present AIKON, a modular platform designed for the study of historical corpora, where users can collaboratively constitute, process and explore digitized documents using computer vision. Among the many tools that have emerged in recent years to empower historians with ai tools, aikon stands out by: (i) providing a comprehensive environment, supporting the historians’ workflow from corpus construction to results validation and visualization; (ii) following a modular and scalable design, that makes it easy to customize the platform and to develop new modules. Fig. 1

Once deployed, aikon guides users through corpus construction, targeted processing using computer vision algorithms, and results exploitation, without requiring any particular technical expertise. In each phase, it emphasizes user control over automatic processing. It handles document privacy, applies selected algorithms to user-defined document sets, and provides interfaces that encourage algorithmic outputs’ validation and correction. Despite providing a comprehensive research environment, aikon is also designed to integrate in larger research processes: it includes diverse import and export functionalities that favor interoperability and enable users to easily share data or leverage aikon outputs through using external tools.

The platform design aims to make it versatile and scalable. It is not conceived as a monolith, but rather as a modular ecosystem where individual processing tasks operate as independent modules within the platform. aikon is thus separated into a core module, which includes a flexible data model to describe documents, corpus management tools, a processing workflow and visualization templates, and optional modules. These optional modules interact with the core module in a standardized way, can implement diverse algorithms, and include specific visualizations. This modular architecture enables decentralized development, encouraging contributors to develop specialized modules and share them with the broader research community. It also aims at increasing the platform’s sustainability, since it can easily incorporate novel tools and algorithms. The platform’s scalability is ensured by technological choices, in particular for data management, and by a separation between the platform itself and an independent gpu-powered api, that can also be leveraged by other applications.

Initially developed with historians of science who focus on the analysis and edition of scientific illustrations, aikon helps them investigate how visual elements contribute to the circulation of scientific knowledge. Detailed use cases of the platform are presented in [1]. Different instances of aikon are used by different teams, integrating different optional modules and different typologies and metadata for the data models. For the need of these projects, algorithms and visualizations have been developed as optional modules for (i) illustration, diagrams, text line and character detection, using the standard yolo [2, 3] and dino-detr models [4, 5], (ii) similar image search using cosine feature similarity for a diverse family of deep image features, as well as the SegSwap algorithm [6] which specifically targets copy detection in historical documents, and (iii) a diagram vectorization algorithm [7]. Historians use the data model to describe manuscripts, woodcut prints and letterpress prints ranging from the 8^th^-18^th^ century, including documents in Sanskrit, Persian, Arabic, Chinese, Greek and Latin, demonstrating its versatility. One of the platforms includes more than 2000 witnesses, containing more than 2.5 million pages, in which the algorithms have identified more than 400 thousand regions among which 4.5 million similarities have been computed, demonstrating aikon’s scalability.

In the rest of the paper, we first review existing computational platforms and tools, clarifying aikon’s positioning, then detail aikon’s workflow, and finally outline aikon’s main implementation and technological choices.

aikon’s open-source code is available on Github https://github.com/Aikon-platform, and two demo versions can be accessed directly from the project’s website https://aikon-platform.github.io/, one of the full platform with restricted rights and accessibility, and a simpler one leveraging aikon’s api to directly run algorithms on custom image sets, without corpus management or results exploitation tools.

**Table 1:** Overview of some digital tools for Historical data processing. See text for details.

Platform	Graphical interface	Custom corpora	Corpus mgmt.	Collab.	Annot. & valid.	iiif	Extensible	Text centered	Available computer vision processing
Kraken [8]		✓						✗	Segment. (page, line), ocr / htr, Training
HTRFlow [9]		✓						✗	Segment. (page, line), ocr / htr
Distant Viewing [10, 11]		✓							Segment. (face), Image analysis, Similarity
pimmi [12]		✓							Similarity
MapReader [13]		✓							Segment. (obj. detection), Training
ArticleSeparation [14]		✓						✗	Segment. (page), ocr
GallicaPix [15]	✓								Search (text)
onit Explorer [16]	✓								Search (text, image)
Visual Contagions [17]	✓								Search (image)
vise [18, 19]	✓	✓							Search (image)
wise[20]	✓	(✓)							Search (multimodal, including audio & video)
eScriptorium [21, 22]	✓	✓	✓	✓	✓	✓		✗	Segment. (page, line), ocr / htr, Training
ArkIndex [23]	✓	✓	✓	✓	✓	✓		(✗)	Segment. (page, table, obj.), Classif., ocr / htr, nlp
campi [24]	✓	✓	✓	✓					Similarity, Search (image)
Panoptic[25]	✓	✓	(✓)		(✓)		✓		Similarity
**AIKON (ours)**	✓	✓	✓	✓	✓	✓	✓		Segment. (illustrations, diagrams, line, character), Similarity,
									Vectorization

## Related works

A large number of digital tools have been developed to handle and process historical document images (see Table 1). However, existing open-source solutions remain limited in scope, often focusing on text processing or image search. On the contrary, aikon aims at providing a complete and generalist research environment. We divided existing solutions into three categories:

- **Processing tools**: command line interfaces providing computer vision algorithms tailored to historical documents
- **Augmented search engines**: interfaces leveraging computer vision for image retrieval without providing specific tools to manage historical corpora
- **Platforms**: complete ecosystems for the management and analysis of historical document collections.

### Processing tools

Command line tools offer low-level and highly adaptable solutions for technically proficient researchers. Some of the earliest and most durable solutions tackle the issue of OCR (Optical Character Recognition) / HTR (Handwritten Text Recognition). Developed continuously since 2015, Kraken [8] has become a staple for historical document processing: it offers layout analysis and character recognition , allowing to process documents with out-of-the-box models, as well as to train custom models. To offer more scalability, the HTRFlow library [9] seeks to streamline the construction of complete pipelines from configuration files.

Beyond text processing, stemming from visual culture studies, the Distant Viewing Toolkit and pimmi aim at processing images in “recent” data, from the 20^th^ century onwards. The Distant Viewing Toolkit [10, 11] offers generic computer vision algorithms such as facial recognition, as well as algorithms for audiovisual data. The pimmi library [12] aim at detecting copies of images in large corpora.

Finally, NewsEye’s ArticleSeparation [14] and MapReader [13] are two libraries offering complete processing chains, each tailored to one type of document – maps and newspapers, respectively. ArticleSeparation focuses on image segmentation, while MapReader is aimed at image classification and offers model training, inference and post-processing capabilities.

These command line tools have the benefit of offering a low-level, hands-on approach to image analysis. However, they are very specialized, either in terms of domain or of available algorithms, while aikon aims to be a more generalist solution. Moreover, the use of these processing tools requires a technical knowledge that humanities researchers often lack.

### Augmented search engines

Computer vision algorithms have been implemented in search engines in order for researchers to be able to query the content of images, rather than their metadata. Released in 2017 by the BnF (Bibliothèque nationale de France), GallicaPix [15], is a notably early implementation of a text-to-image search engine on the BnF catalog. vise [18] developed by the University of Oxford’s VGG (Visual Geometry Group), functions as an image-to-image search engine and has been popular among humanity researchers, since it enables installing a local version to search ones own database. Visual Contagion’s Explore [17] also provides image retrieval tools, but is notable for extending the search engine paradigm with image clustering, corpora filtering and data export features.

In 2021, the development of clip [26] enabled the use of generalized deep multi-modal models for image-to-image and text-to-image search without training custom models. The onit Explorer [16] employs clip features as a common representation space enabling both image and text queries for exploring curated collections of images from travelogues. The VGG’s wise platform expands on the functionalities offered by vise and implements multi-modal search on image and audio-visual data: text and image-to-image search (using clip), audio content search, face recognition. Notably, wise can be expanded by implementing new feature extractors.

While these computer vision augmented search engines are a solution to bypass the lengthy metadata creation process and offer appealing results, they have several limitations. First, they follow an ETL (Extract-Transform-Load) paradigm [15], and feature extraction typically operates on predefined corpora prior to deployment, limiting flexibility for corpus modifications without technical knowledge. Second, they are mostly limited to image content search. Third, they offer limited corpora management and collaboration possibilities.

**Fig. 2:**
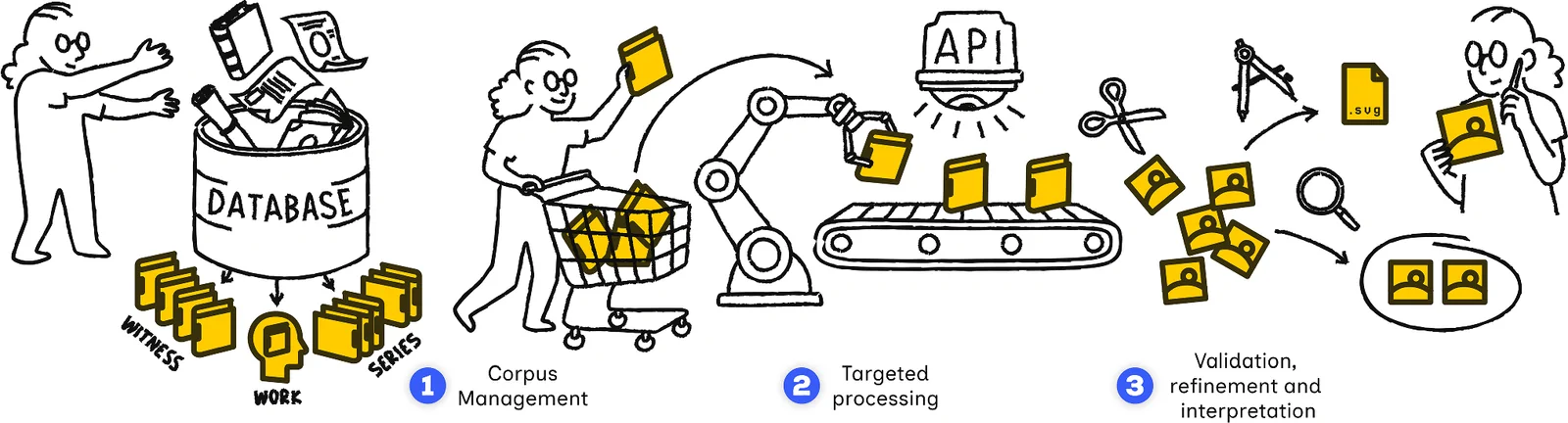
aikon guides users through a three step workflow

### Platforms

We call “platforms” tools that offer corpus management and collaboration tools via a graphical interface requiring no technical knowledge.

Some platforms such as Heurist [27] and Omeka [28] do not provide any computer vision processing functionalities. In this category, some focus on collaborative manual annotation, such as Arvest [29] which leverages IIIF (International Image Interoperability Framework) video annotation for the documentation of live performances, or Callico [30] and FromThePage [31] which focus on text transcription.

In the 2020s, several platforms have emerged to carry out computer vision tasks. eScriptorium [21, 22] and ArkIndex [23] focus on text recognition. eScriptorium offers a graphical user interface to Kraken processes. ArkIndex is arguably the closest platform to aikon: it provides a multi-user environment to perform a wide array of deep learning algorithms on historical documents. However, where ArkIndex focuses primarily on document processing workflows, aikon emphasizes exploratory analysis, with integrated tools for interpretation and visualization. campi [24] and Panoptic [25] are to examples of platforms that do not focus on text. campi [24] is a multi-user collection management system that relies on image similarities to speed up metadata creation on large corpora: groups of similar images can be annotated together. Panoptic [25] is focused on similarity computation. Aimed at individual researchers, it can only be used locally and provides similarity and clustering algorithms, as well as metadata edition interfaces.

aikon falls into this platform category, aiming at providing a complete environment for the management and analysis of historical corpora. Similar to ArkIndex, Arvest and Callico, it deeply integrated with IIIF technologies; much like Omeka and Panoptic, it aims to foster extensibility and a modular design philosophy.

## AIKON workflow

aikon provides a comprehensive ecosystem covering an entire workflow of visual analysis for historical corpora, visualized in Figure 2. It aims at enabling researchers to scale from focused individual studies to comprehensive cross-collection collaborative investigations. This workflow and the associated choices are at the core of the platform’s technical design, described in Section 4. They aim at being general, flexible, and easily adaptable to different types of corpora, tasks and analysis.

aikon guides users through three interconnected phases, which we detail in the next subsections, each addressing a distinct component of the research workflow:

- **Corpus construction and management**: Users upload digitized documents, transforming scattered sources into a coherent collection organized through a flexible data model that preserves contextual relationships across documents.
- **Targeted processing**: From the assembled corpus, users select specific subsets for tasks such as extraction of document regions, identification of similar regions, and region processing, which can be performed manually or using computer vision algorithms.
- **Validation, refinement and analysis**: Processing outputs are accessible through visualization interfaces designed for scholarly investigation. Integrated annotation and correction tools allow researchers to refine and contextualize results, while export features facilitate integration with external analytical tools.

### Corpus management

aikon balances two complementary objectives: preserving the complexity of heritage collections and their descriptions, while structuring data for algorithmic processing. To do so, we developed a data model relying on six core entities, which we present first. We then discuss in more detail how the *Regions* entity is used for visual annotation and processing. Finally, we explain how we ensure interoperability through the consistent use of the IIIF framework.

#### Core entities

**Fig. 3:**
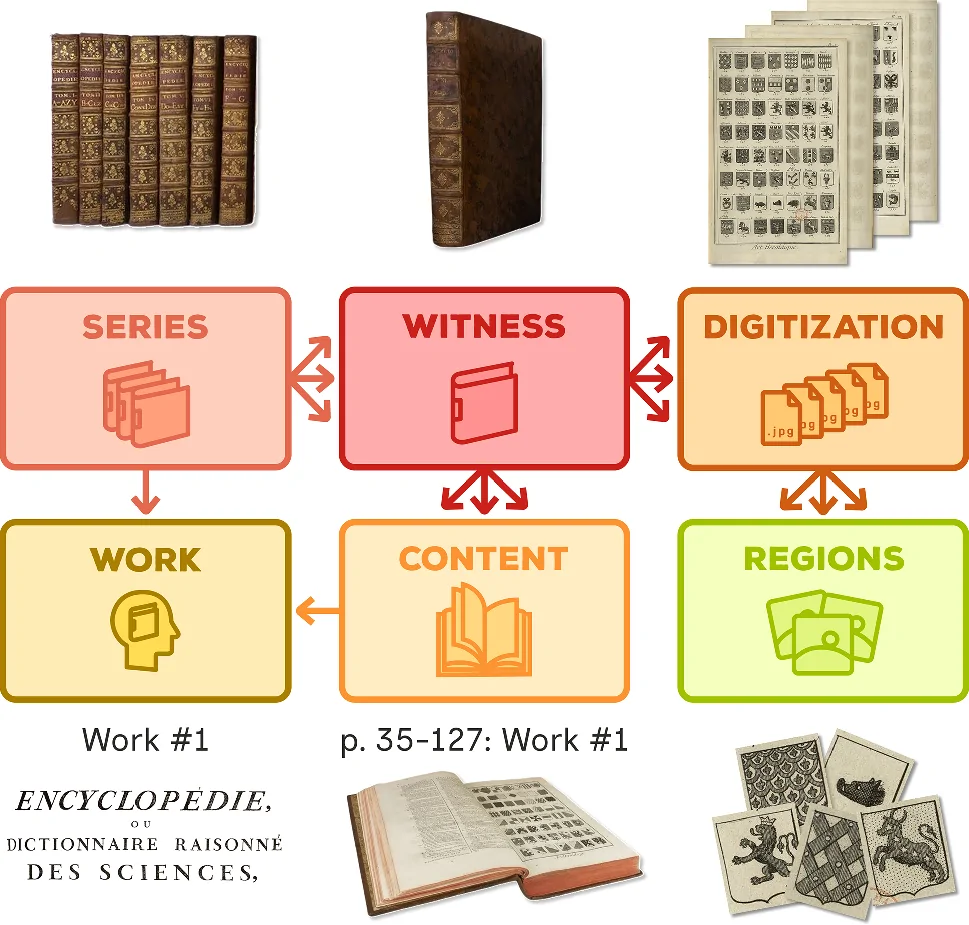
Core database entities and their relationship exemplified with the *Encyclopédie* of Diderot and D’Alembert. These entities can be associated to different typologies, depending on the type of historical artifacts they describe

aikon’s data model distinguishes intellectual content from physical materiality, enabling researchers to map connections and transmission dynamics within scattered corpora. Inspired by CIDOC CRM (CIDOC Conceptual Reference Model) and FRBR (Functional Requirements for Bibliographic Records) standards, it defines the following core entities, visualized in Figure 3:

1. *Work*: represents the abstract intellectual production, encompassing the core ideas and content across various material incarnations.
2. *Witness*: denotes a distinct physical object or primary source preserved at a specific location.
3. *Series*: encompasses a group of witnesses sharing a common coherence, such as volumes of an edition or manuscript fragments held in different institutions.
4. *Content*: embodies the specific manifestation of a work within a witness.
5. *Digitization*: Digitizations are attached to witnesses, allowing for direct association between the physical object and its digital representation(s).
6. *Regions*: consistent set of image regions extracted from a digitization.

**Fig. 4:**
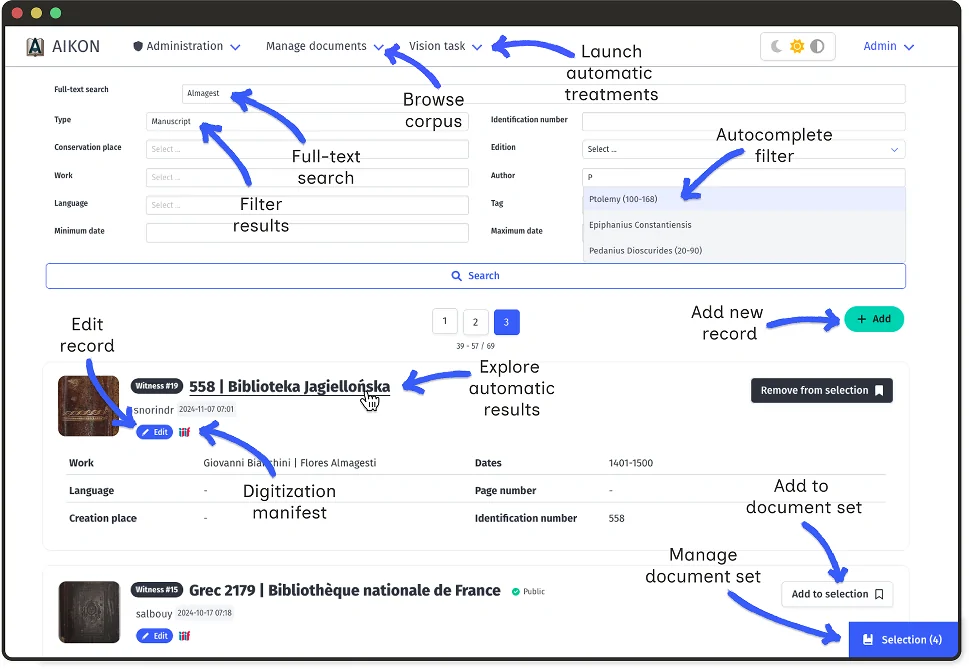
aikon enables creating a research corpus, searching and filtering it through metadata, and creating sets of relevant documents for specific research questions

This structure allows for a nuanced representation of complex contents, particularly useful for objects such as manuscripts where multiple distinct works may be intertwined within a single physical document. It also enables to associate metadata to the different entities, organizing the corpus and making it searchable (see Figure 4). aikon currently supports three documentary typologies: manuscripts, letterpress prints, and woodblock prints. Each typology defines specific nomenclature and associated metadata fields, such as publisher details for letterpress prints. New typologies can easily be integrated to fit different kinds of materials. Researchers can of course fill the metadata at various levels of details depending on their goal, from detailed metadata description for in-depth investigations to streamlined document upload for exploratory analysis. Access restrictions to digitizations and editorial rights are managed through dedicated metadata fields and user group assignments.

#### Core visual unit: *Regions*

Image regions form the core units of visual analysis. An image region is defined through its coordinates within a digitization page. They are structured within the *Regions* entities that correspond to the complete set of regions extracted by a particular method–automatic or manual–from a single digitization. Each *Digitization* can be linked to multiple *Regions* entities, typically corresponding to different types of visual elements. This design also enables comparative analysis across different automated localization approaches while maintaining methodological traceability. Regions then serve as the basis for subsequent processing, such as HTR on text lines, vectorization on diagrams, or similarity search on illustrations. To represent similarity between regions, the *RegionPair* relationship records comparative data between two image regions, such as automatic similarity scores or researcher-assigned categories. Image region, *Regions*, and *RegionPair* entities are the foundation for aikon’s cross-corpus analysis.

#### Export, imports and interoperability

aikon integrates the IIIF standard as the foundation of its visual resource management, serving the dual objective of ensuring interoperability with existing heritage ecosystems and providing researchers with advanced tools for image manipulation. Imported digitizations are transformed into IIIF manifests, to provide a consistent interface for visualization, sharing, and annotation.

Our *Regions* entities are defined by IIIF annotations, used for manual exploration and automated computer vision processing. This system preserves file integrity by avoiding fragment duplication, and keeps all annotations contextually linked. Generated manifests and their associated annotations become portable assets that researchers can integrate into any external IIIF-compliant solution.

### Targeted processing

**Fig. 5:**
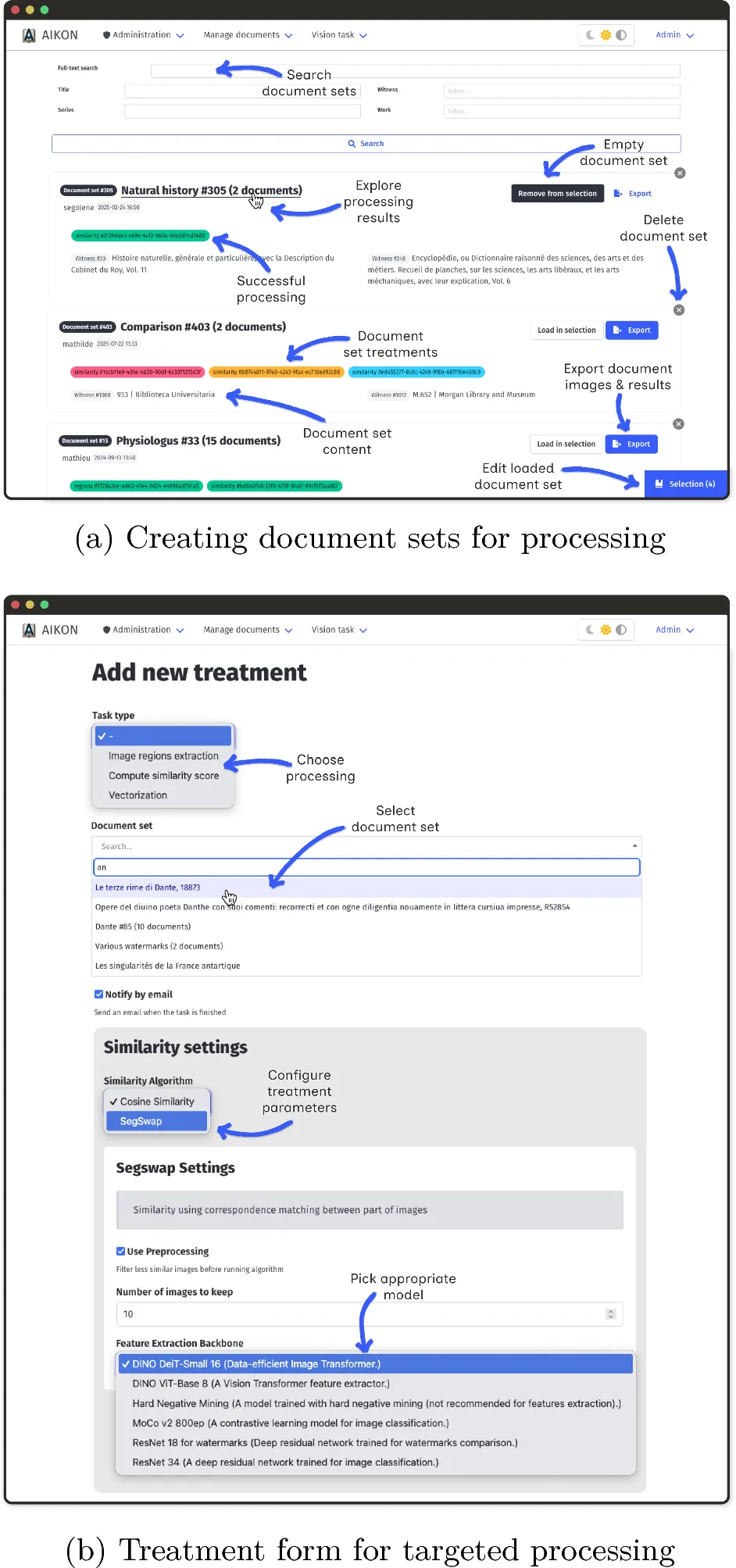
Users select sources to create a document set (5a), then configure task parameters with the treatment form (5b) to launch automatic processing using diverse computer vision algorithms

To facilitate intellectual organization and algorithmic processing within large collections, aikon enables researchers to create document sets that group materials according to their specific logic. This is also required for scalability, because aikon gives access to many algorithms that would be prohibitively costly to run across a complete corpus. Figure 5a shows how document sets can be created from the search interface, which also list past processing results, and Figure 5b shows how processing can be launched on a document set with specific algorithm parameters. In the rest of this section, we detail the different types of processing that aikon supports, the modular implementation principle that enables to easily add new algorithms, and already implemented algorithms.

#### Processing typology

aikon handles three types of processes, which can be executed using automated algorithms or manual annotation workflows. They can all be applied to document sets but differ by their output, and correspond to distinct approaches to source analysis:

- *Region localization* processes identify visual components within documents, such as illustrations or text lines. This category encompasses tasks such as layout analysis, page segmentation, or object detection. Results are stored as *Regions* entities that can serve as base for subsequent analysis.
- *Region comparison* processes establish connections between localized regions. Algorithms will typically output similarity scores for pairs of regions. They aim at enabling researchers to study the circulation of visual motifs across extensive corpora.
- *Region analysis* processes focus on each region independently to generate additional metadata, such as tags, vectorization or transcription.

#### Modular implementation

All processes are implemented as independent modules that operate within a common structural framework. More precisely, they are associated to parallel modules implemented both in the front-end application and in the api – a program that receives requests from the web platform and returns results, typically the output of computer vision algorithms. Each module functions as a self-contained processing unit, receiving input data and task parameters, executing specialized algorithms through api calls, and returning formatted results displayed through dedicated interfaces. This separation ensures that modules can be enabled or disabled without affecting other platform components.

**Fig. 6:**
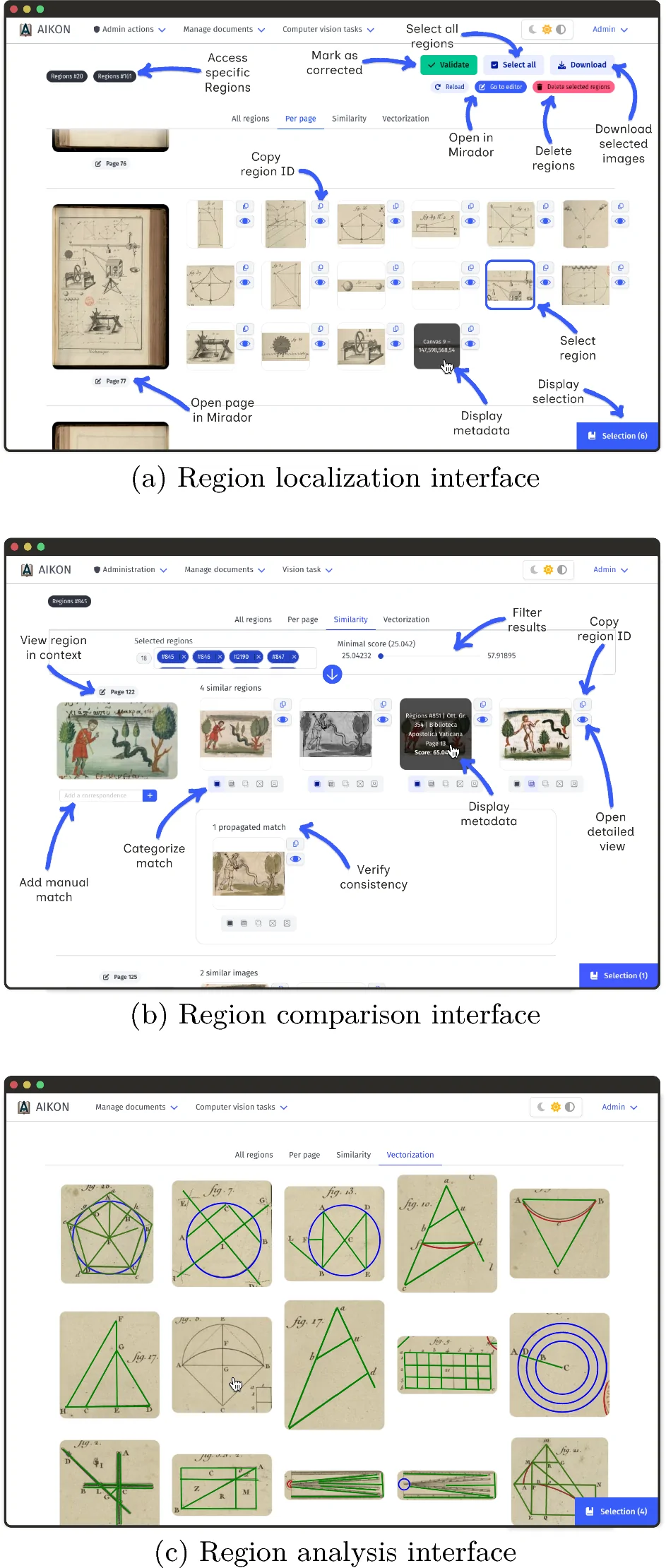
Our document-level interface includes visualizations associated to our three main treatment types, regions extraction, comparison and analysis. It aims at easing and encouraging validation and correction for a specific document

These modules operate within a comprehensive processing environment, defined in the aikon core module, that manages task execution, process configuration, and result manipulation. It maintains full traceability of processing parameters to ensure reproducibility, while a centralized monitoring system coordinates operations across the api and provides user notifications. The current module set is designed for extensibility, allowing new modules to build upon existing components while adding specialized functionality. Our goal is to build a collaborative ecosystem where contributors can easily add new computational methods.

#### Implemented algorithms

The current aikon platform already implements several algorithms. For region localization, it includes approaches based on yolo [2] and dino-detr [4] detection. We adapted these algorithms and trained them on specific corpora to detect illustrations and astronomical diagrams following [3] as well as text lines and characters following [5]. Region similarity computation algorithms include cosine similarity with a diversity of deep features [32–35] as well as the SegSwap [6] algorithm that targets exact visual repetitions, making it suitable for detecting copied patterns, while cosine similarity typically captures broader semantic correspondences across images. Finally, as an example of analysis algorithm, we implemented diagram vectorization  [7]. We also plan to integrate OCR/ HTR as a region analysis tool (as opposed to the current character detection tool) in the near future.

### Validation, refinement and analysis

**Fig. 7:**
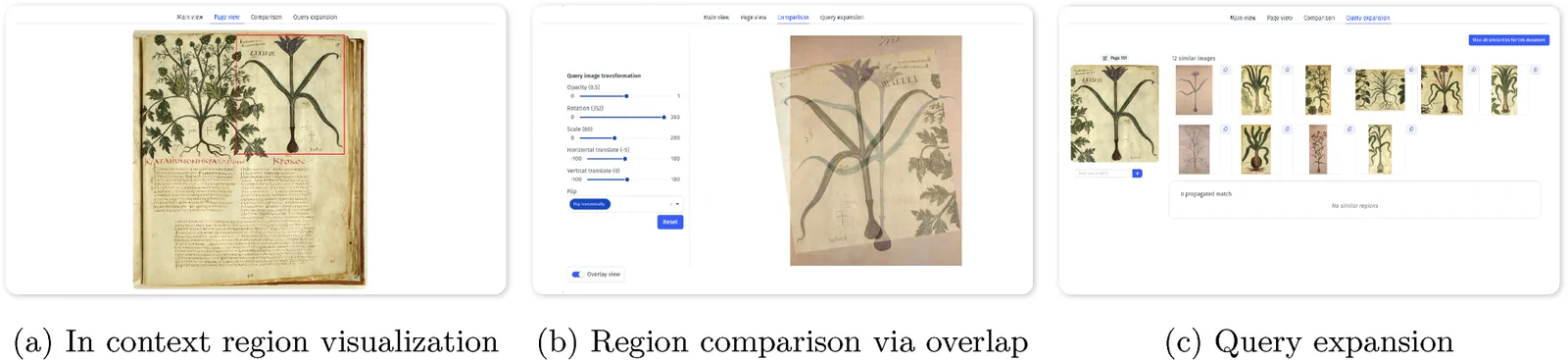
Our region-level interface enables a detailed view with a focus on a specific region, visualizing it in context (7a), comparing it to a matched region either side-by-side or through overlapping (7b ), and using it for query expansion, visualizing the other regions it is matched to (7c)

**Fig. 8:**
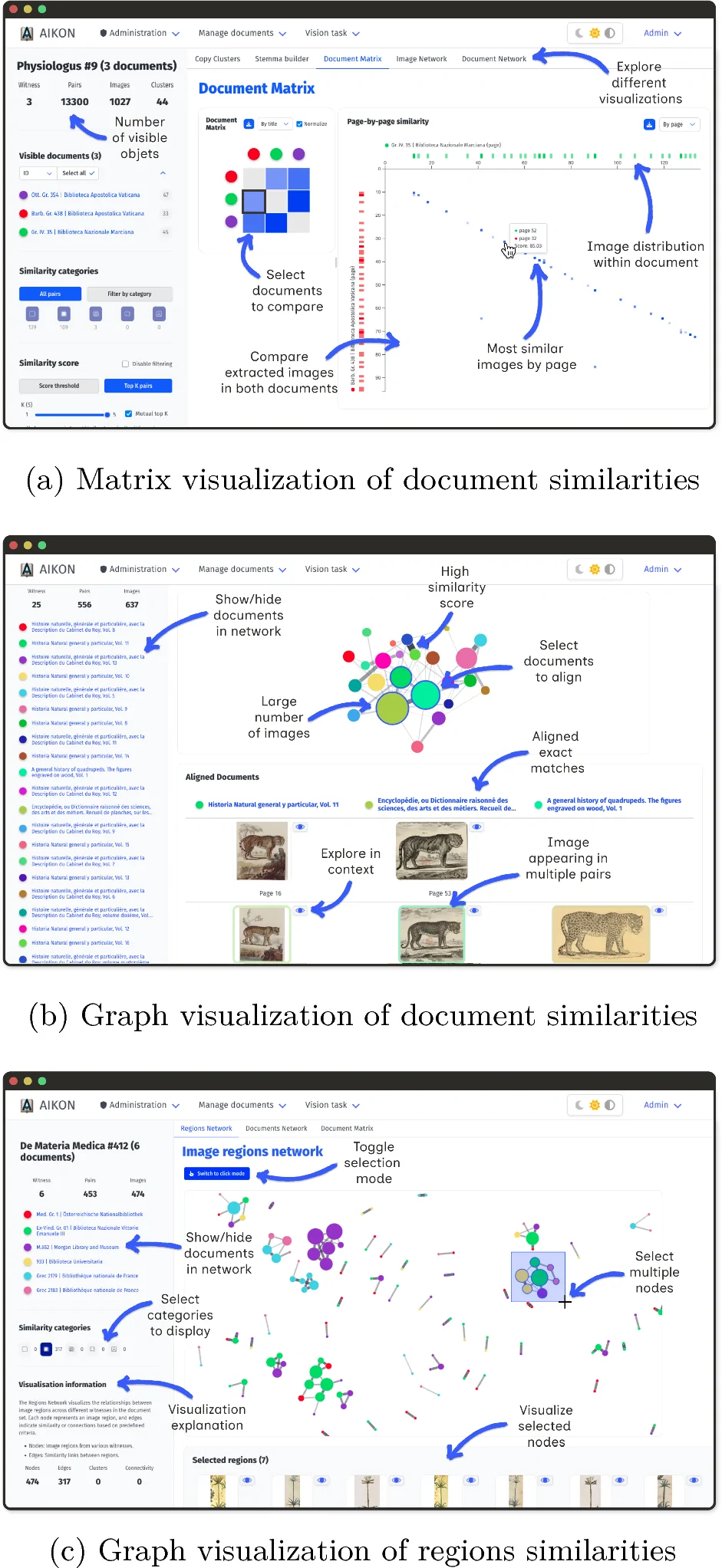
Our document set-level interfaces enable to analyze similarity networks at different levels: complete documents (8b and 8a) or individual regions (8c)

aikon’s visualization and editing interfaces aim at maintaining researcher’s control over algorithmic results, encourage systematic validation and correction, and enable critical examination. In the next sections, we present the interfaces associated to a document (Section 3.3.1,  Figure 6) a specific region (Section 3.3.2,  Figure 7) or a document set (Section 3.3.3, Figure 8). Moreover, because we want aikon to be easily integrated with other analysis tools, it includes export options described in Section 3.3.4.

#### Document-level interface

To explore regions localized in a document, users can alternate between two views: a document overview that enables quick identification and removal of irrelevant regions, and a page-based view shown in Figure 6a. Both interfaces enable direct access to a Mirador viewer [36], where researchers can refine bounding boxes, remove erroneous detections, or add missed elements.

The region comparison interface, shown in Figure 6b, displays for all regions localized in a document ranked matches for each region across selected documents, enabling researchers to classify correspondences in a single click (as an exact, partial, semantic, or user-defined match). They can also copy, in a single click and from any view, the id of a specific region and add it as a match to any other region. The annotated matches are used for match propagation – if A matches B and B matches C, A is inferred to match C – creating expert-validated similarity networks that facilitate discovery while revealing annotation inconsistencies.

The region analysis interface (Figure 6c) currently only enables vectorization results visualization, but we plan to add interfaces for image level classification and text transcription, as well as tools for validation and correction.

#### Region-level interface

To enable user to focus on a specific region, each region is associated to a detailed view, shown in Figure 7. It features in-page contextualization (Figure 7a), a region comparison tool that enables overlaying associated regions with rotation and positioning controls (Figure 7b), and a query expansion window where the regions becomes the origin of a search for matching regions (Figure 7c).

#### Set-level interface

To support the analysis of historical transmission networks across document sets, aikon provides matrix-based and graph-based visualizations. These visualizations leverage both automatic similarity scores and user-validated categorizations to analyze historical transmission at different levels of granularity. Filters by document or correspondence type are available in all visualizations.

At the document level, the interface aggregates region-level similarities between documents.

It first provides matrix-based visualizations for pairwise document comparisons (Figure 8a). A similarity matrix between selected documents (left) enables to quickly identify visual overlap between documents through color intensity, and thus groups of highly related documents. By clicking on a particular point of this first matrix, corresponding to a pair of documents, one opens another matrix visualization (right) for this pair of documents, similar to the ones presented for image collation in [37]. The axes correspond to the ordered illustrations in each document and the intensities to similarity rank or annotations. These visualizations help researchers identify whether copied illustrations maintain their original order, appear clustered in specific sections, or follow a reorganization patterns.

At document level, the interface also proposes a graph-based visualization (Figure 8b), where nodes represent documents. The edges’ width correspond to the number of associated regions between two documents, and the node size to the connectivity of the associated document. This view enables researchers to identify documents with many connections and to examine the nature of these connections. Selecting document nodes displays at the bottom a table of all connected regions, making it easy to identify visual shifts, such as systematic mirroring of the illustrations.

Finally, at the region level, the interface displays all extracted regions within a document set as a network, where nodes represent individual regions and edges represent correspondences between regions (Figure 8c). The node’s size is are proportional to the number of connections of the associated region, while the edge’s width reflects the degree of similarity between connected regions in case a similarity score is available. Selecting nodes provides direct access to the corresponding images and their metadata. This enables to easily identify unique or very common illustrations.

#### Exports

Beyond integrated visualization tools, aikon is designed to serve as a component of a broader digital humanities ecosystem, rather than a standalone tool, and thus incorporates comprehensive export options. A restful api delivers structured metadata and results in json, while zip archives bundle digitized images, structured metadata, and results in standard formats. These capabilities allow researchers to integrate aikon’s outputs with their own analytical methods, whether qualitative or quantitative, maintaining maximum interoperability.

#### Annotation efficiency evaluation

**Table 2:** Comparison between manual and semi-automatic region extraction. Time is in minutes:seconds and A1-3 refers to the annotators.

	Manual			Semi-automatic		
	A1	A2	A3	A1	A2	A3
**G. 1551**						
Time	31:44	38:11	18:45	3:04	5:58	7:09
F1	0.95	0.93	0.99	0.99	0.91	0.99
**S. 1838**						
Time	35:22	62:30	32:08	7:35	17:10	12:13
F1	0.97	0.80	0.98	0.93	0.96	0.94
Avg.	36:27 / 0.94			08:52 / 0.95		
Time/F1						

To evaluate the practical benefit of aikon’s processing tools, we compared two workflows for region extraction: fully manual annotation in a Mirador viewer, and semi-automatic extraction using models from the platform followed by manual correction.

Three annotators annotated two documents using both workflows:

- *Gesner 1551*[38], an early print containing 142 illustrations across 507 pages;
- *Sprenger 1838*[39], an Arabic astronomical manuscript containing 241 diagrams across 337 pages.

We report in Table 2 execution time and F1 score, using an IoU threshold of ≥0.5, against a ground truth defined using all annotations. The semi-automatic and manual workflow achieves on average comparable F1 scores. Differences in F1 scores mainly reflect annotator choices rather than errors: some group adjacent illustrations into one bounding box while others separate them.

For all annotators, the automatic workflow reduces significantly annotation time, while achieving scores comparable to manual annotation. Moreover, annotators consistently reported semi-automatic annotation was much less demanding, because much less clicks and bounding box drawing were necessary.

## AIKON implementation

**Fig. 9:**
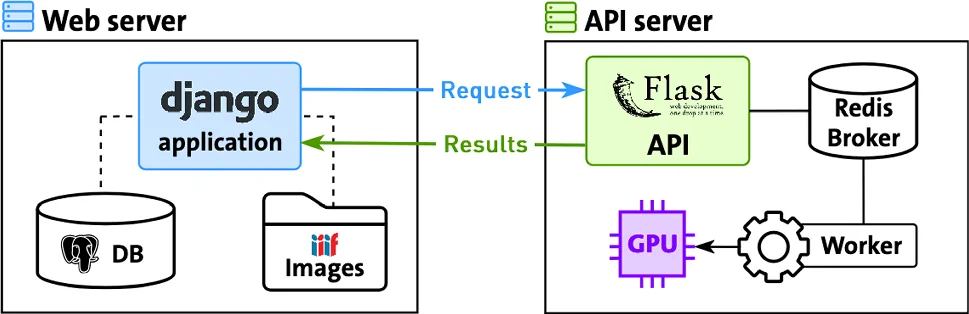
Interactions between application and api

This section presents aikon’s implementation and key technological choices, summarized in Table 3. On a high level, aikon’s architecture relies on interactions between an application and an api that can be deployed on either the same or different servers, as visualized in Figure 9. These two main components interact through http request exchanges, with the platform storing images, data and results, and the api focusing on the application of computer vision algorithms.

aikon’s design is modular, so that it can easily be adapted to any specific research project’s needs, and be extended to incorporate novel tools. We first discuss the core module in Section 4.1, then optional modules in Section 4.2 and finally deployment in Section 4.3.

**Table 3:** Overview of technologies used by aikon.

Role	Technology
Images	IIIF
Image server	Cantaloupe
Annotation server	aiiinotate
Application	Django
Database	PostgreSQL
User interface	Svelte , Bulma
Tasks management	Celery , Redis
API	Flask
Tasks management	Dramatiq , Redis
Deployment	Docker

### Core module

aikon’s core module is the base of the application and encompasses essential services such as data, tasks and user management, as well as the skeleton for the interfaces, including templates that can be extended in optional modules. It was designed to provide users with a swift experience even when dealing with very large databases. In this section, we describe our choices for this module, including image management, application, and api.

#### Image management

aikon relies on the IIIF framework and ecosystem for image management. This standard was designed to facilitate access to heritage images while enabling image manipulation and interoperability, and it has been widely adopted by heritage institutions and digital humanities tools.

All digitizations imported in the platform, in PDF or as image files, are automatically converted into IIIF manifests, turning heterogeneous image files into interoperable resources.

To handle its IIIF image database, the aikon core module incorporates Cantaloupe [40], a IIIF compliant image server, along with aiiinotate [41], a custom IIIF annotation server used to store and manipulate image regions. aikon also incorporates a Mirador viewer [36], which enables both advanced visualization and annotation editing.

We found it necessary to develop our own annotation server, **aiiinotate ** [41], because aikon’s use of IIIF to handle massive quantities of algorithmically produced data led to bottlenecks with existing annotation servers. For example, SAS (Simple Annotation Server) [42], which we used in a preliminary version of the platform, lacks features for bulk operations (e.g., inserting all annotations generated by a *Regions* extraction in a single query) and was the main cause of latency in aikon . Built with NodeJS-based [43] api framework Fastify [44], aiiinotate stores annotations in a MongoDB database [45]. We leverage NodeJS’ non-blocking I/O and Fastify’s asynchronous architecture to serve multiple clients concurrently, and use MongoDB indexes to increase the speed of database queries. We performed the following scalability test on aiiinotate: we first created a database of 10.000 documents with 1,000 pages each, and 1,000 annotations on a random subset of 1% of the pages, leading to 100 million annotations; we then queried the server with 20 concurrent clients for all annotations in a specific page. The annotation server did not saturate and returned the 1,000 annotations in 0.02 seconds on average, demonstrating its scalability.

#### Web application

The aikon platform is built using Django [46], a Python framework for application development providing security features, along with various tools for user management (accounts, groups and permissions), requests handling and database integration. It uses a PostgreSQL [47] database system which, linked with Django, implements the aikon data model.

Queues and task scheduling are handled by Celery [48] and Redis [49]. Celery leverages the multiprocessing functionalities of the application by running asynchronous tasks in the background. It ensures an optimized workflow for time-consuming tasks, such as image conversion, json generation and annotation parsing.

For its interfaces, aikon relies on the JavaScript framework Svelte [50], designed for efficiency and responsiveness. In the platform, list interfaces handle the dynamic display of database entries.

It also leverages Bulma [51], a lightweight css framework selected for its easily customizable elements and predefined classes for page layout and presentation.

#### API

aikon api is developed to be independent from the aikon platform, with its own technical specifications. It can therefore be leveraged by other applications. The api is designed to be deployed on a gpu server, providing the computational power required by computer vision algorithms, and connected to the platform through http requests.

aikon api is built using Flask [52], a lightweight Python web framework, with Dramatiq [53] as a queue manager. Dramatiq handles parallelism to avoid overloading, manages image downloading as a background task, and launches the heavy computations some tasks require.

The core api module provides essential endpoints for task management, and can be extended with modules for each computer vision task, which rely on those endpoints. The api is not developed to store or display data and images, and therefore has no front-end for users to interact with.

### Optional modules

To make aikon a versatile and flexible platform that can be tuned to the need of different projects and can follow the evolution of algorithms, most treatments and interfaces are implemented as optional modules, that can be selected or not during platform deployment.

#### Existing modules

The current aikon platform includes examples of modules for the three types of processing described in Section 3.2. Each includes an application module, which adapts the treatment form and adds new visualization interfaces, and an api module, with specific inputs, processing and outputs. On the application side, interfaces are shown in Figure 6. On the api side:

- the region localization module receives a list of IIIF manifests of complete digitizations of sources. It stores them temporarily, runs the detection algorithm on each image, and saves the regions in a json file that is returned to the platform.
- the region comparison module receives a list of region images to download for each witness, compares every region in pairs and returns the results in a json file with scores for selected image pairs.
- as an example of region analysis modules, we implemented a diagram vectorization algorithm, which receives as its input a list of regions, downloads them, runs the vectorization algorithm, and returns a zip file containing svg files for each region.

Inference for region extraction and similarity retrieval can be run on cpu, while vectorization requires the api to be deployed on a gpu.

#### Developing a new module

To add a novel computer vision algorithm to the platform, symmetrical modules need to be developed for the web application and the api. Modules are Python packages with a set of standard files and folders, some mandatory and some optional. On both the api and application side, similar files define the module tasks and URL routing. On the application side, additional files define, for example, the adaptation of the treatment form, extensions of the data model or new interfaces. On the api side the module also includes the computer vision algorithms.

### Deployment

The aikon platform uses Gunicorn [54] as a wsgi server with Nginx [55] as its http server to serve the application on the web.

aikon relies on Docker [56] for deployment^1^, to ease package installation and updates through containerization. A deployed instance of aikon combines eight Docker containers for the application, database, Cantaloupe server, annotations management and Nginx, connected through a network and shared data folders.

aikon provides a script to restart, stop or build Docker containers to ease the troubleshooting process, and to apply updates pulled from the remote repository. Deployment can also be performed without Docker^2^ using Gunicorn and Nginx, with a manual set up and management of environments and packages.

The api is also deployed with Docker, with the option to used spiped [57] to secure connection. It can be deployed independently from the platform on a gpu server, or on the same server in its specific container.

During deployment of aikon, modules are installed automatically through environment variables, which allow projects to select specific tasks and related tools to be used in production.

### Limitations

The current state of the platform has limitations both in terms of sources it can handle and implementation.

In terms of sources, we identify three main limitations. First, aikon is geared towards digitized textual documents and does not handle raw text, audio, video, or other media. Second, it does not yet integrate specialized processing for specific document types (e.g., maps, newspapers) or edge cases such as heavily degraded documents. Third, the available algorithms are centered around illustrations, and OCR / HTR / NLP (Natural Language Processing) modules have not been implemented yet. Nonetheless, aikon’s modular architecture is designed to allow new modules and models to be developed to address these limitations.

In terms of implementation, we identify two main limitations. First, only Linux and Mac operating systems are currently fully supported. Second, for its image processing, aikon still relies on the IIIF Presentation API 2.1, although the IIIF Consortium has introduced IIIF Presentation API 3.0, whose adoption remains however limited.

## Summary

aikon addresses the needs of historians working with visual materials by providing a unified platform that connects computer vision technologies with humanities research practices. The platform enables researchers to conduct systematic analysis of historical corpora while maintaining control over their sources and analytical processes. By combining flexible corpus management, automated processing capabilities, and comprehensive result validation tools, aikon creates an environment where historians can leverage computational methods without requiring extensive technical expertise.

Through its standardized framework for integrating new modules, aikon promotes the creation of a shared ecosystem where individual project developments benefit the broader research community. This modular approach accommodates current research needs alongside long-term sustainability while aiming at promoting collaborations in the implementation of artificial intelligence in humanities research.

Concrete use-cases of aikon for historical research are discussed in [1].

## Data Availability

No datasets were generated or analysed during the current study.

## Footnotes

1 Documentation on Docker configuration for deployment can be found on aikon’s wiki, with setup requirements and troubleshooting information. https://github.com/Aikon-platform/aikon/wiki/Docker-deploy

2 Documentation for a docker-free deploy can be found here: https://github.com/Aikon-platform/aikon/wiki/Deploy-without-Docker

## Declarations

### Code availability

aikon’s code base is entirely open source. The code of the platform (github.com/Aikon-platform/aikon), its API (github.com/Aikon-platform/aikon-api) and aiiinotate (github.com/Aikon-platform/aiiinotate) are hosted on GitHub.
